# High-level expression of thermostable cellulolytic enzymes in tobacco transplastomic plants and their use in hydrolysis of an industrially pretreated *Arundo donax* L. biomass

**DOI:** 10.1186/s13068-016-0569-z

**Published:** 2016-07-22

**Authors:** Daniela Castiglia, Lorenza Sannino, Loredana Marcolongo, Elena Ionata, Rachele Tamburino, Angelo De Stradis, Beatrice Cobucci-Ponzano, Marco Moracci, Francesco La Cara, Nunzia Scotti

**Affiliations:** CNR-IBBR UOS Portici, National Research Council of Italy, Institute of Biosciences and BioResources, Research Division Portici, Via Università 133, 80055 Portici, NA Italy; CNR-IBBR UOS Naples, National Research Council of Italy, Institute of Biosciences and BioResources, Research Division Naples, Via P. Castellino 111, Naples, Italy; CNR-IBAF UOS Napoli, National Research Council of Italy, Institute of Agro-environmental and Forest Biology, Research Division Naples, Via P. Castellino 111, Naples, Italy; CNR-IPSP UOS Bari, National Research Council of Italy, Institute for Sustainable Plant Protection, Research Division Bari, Via Amendola 165/A, 70126 Bari, Italy

**Keywords:** Green factory, Plastid transformation, (Hemi)cellulolytic enzymes, Bioconversion, Industrially pretreated plant biomass

## Abstract

**Background:**

Biofuels production from plant biomasses is a complex multi-step process with important economic burdens. Several biotechnological approaches have been pursued to reduce biofuels production costs. The aim of the present study was to explore the production in tobacco plastome of three genes encoding (hemi)cellulolytic enzymes from thermophilic and hyperthermophilic bacterium and *Archaea*, respectively, and test their application in the bioconversion of an important industrially pretreated biomass feedstock (*A. donax*) for production of second-generation biofuels.

**Results:**

The selected enzymes, endoglucanase, endo-β-1,4-xylanase and β-glucosidase, were expressed in tobacco plastome with a protein yield range from 2 % to more than 75 % of total soluble proteins (TSP). The accumulation of endoglucanase (up to 2 % TSP) gave altered plant phenotypes whose severity was directly linked to the enzyme yield. The most severe seedling-lethal phenotype was due to the impairment of plastid development associated to the binding of endoglucanase protein to thylakoids. Endo-β-1,4-xylanase and β-glucosidase, produced at very high level without detrimental effects on plant development, were enriched (fourfold) by heat treatment (105.4 and 255.4 U/mg, respectively). Both plastid-derived biocatalysts retained the main features of the native or recombinantly expressed enzymes with interesting differences. Plastid-derived xylanase and β-glucosidase resulted more thermophilic than the *E. coli* recombinant and native counterpart, respectively. Bioconversion experiments, carried out at 50 and 60 °C, demonstrated that plastid-derived enzymes were able to hydrolyse an industrially pretreated giant reed biomass. In particular, the replacement of commercial enzyme with plastid-derived xylanase, at 60 °C, produced an increase of both xylose recovery and hydrolysis rate; whereas the replacement of both xylanase and β-glucosidase produced glucose levels similar to those observed with the commercial cocktails, and xylose yields always higher in the whole 24–72 h range.

**Conclusions:**

The very high production level of thermophilic and hyperthermophilic enzymes, their stability and bioconversion efficiencies described in this study demonstrate that plastid transformation represents a real cost-effective production platform for cellulolytic enzymes.

**Electronic supplementary material:**

The online version of this article (doi:10.1186/s13068-016-0569-z) contains supplementary material, which is available to authorized users.

## Background

In last years, the increased demand for energy and the need for sustainable production of alternative fuels redirected the research priorities toward the use of plant biomasses as a cost-effective and abundant source of renewable energy. Recently, *Arundo donax* L., common name giant reed, has been proposed as feedstock to develop biorefinery because, compared to other energy crops, it is adaptable to different environments, soils and growth condition, requires low input for its cultivation, and is characterized by high biomass production. Further, it has been reported a higher bioethanol production from giant reed than other energy crops [1]. Lignocellulosic plant biomasses consist mainly of three types of polymers, lignin, cellulose and hemicellulose, interlinked in a hetero-matrix, which abundance varies in different types of biomass [2–6]. Biofuels production from plant biomasses is a complex multi-step process that includes collection and transport of the lignocellulosic materials, pretreatments to remove lignin, cellulose conversion into fermentable sugars, fermentation and distillation of ethanol [4, 5]. Important economic burdens of this multi-step process are the costs of thermal and/or chemical pretreatments, and of production and the high dosage of cellulolytic enzyme cocktails required for cellulose conversion [4, 6, 7]. To reduce these costs, several biotechnological strategies have been pursued, such as the modification of lignin content or composition to change the cell wall recalcitrance [8, 9], the genetic engineering of existing fermentations strains to enhance saccharification and fermentation abilities [10, 11], an *in planta* approach for heterologous expression of genes encoding cellulolytic enzymes to avoid exogenous enzyme addition prior to fermentation [12, 13], and the use of heterologous systems as ‘biofactories’ for cost-effective production of cellulolytic enzymes [14–25]. Among them, the recombinant production of cellulolytic enzymes by a wide range of heterologous expression systems (e.g., bacteria, yeasts and plants) has had a huge boost over past decades, and plants represent the most promising system to produce these recombinant proteins due to low production costs and because several strategies have been developed to overcome main limitations [26]. In particular, chloroplast transformation technology shows some attractive advantages (e.g., site-specific gene integration, possibility to express multiple genes arranged as operon, transgene containment due to maternal inheritance of plastid in most crops) and allowed extraordinarily high accumulation levels (up to 70 % of total soluble proteins, TSP) for different foreign proteins [27–29]. Further, it has been demonstrated that chloroplast is also a suitable site for the expression of various cellulolytic enzymes from mesophilic and thermophilic fungi and bacteria [15, 19, 23, 24, 30–36]. Since the existing enzymatic hydrolysis, carried out at ≤50 °C, shows some limitations, such as slow enzymatic hydrolysis rate and low yields of sugars, their overcoming has been proposed using highly thermostable enzymes isolated by thermophilic or hyperthermophilic bacteria [7]. To date, only one hyperthermophilic cellulase was produced in transgenic chloroplasts [18].

The aim of the present study was to explore the production in tobacco plastome of three genes encoding (hemi)cellulolytic enzymes (*endo*-*β*-*1,4*-*xylanase*, *endoglucanase* and *β*-*glucosidase*) isolated from a thermophilic bacterium (*Alicyclobacillus acidocaldarius*) and two hyperthermophilic species of *Archaea* (*Sulfolobus solfataricus* and *Pyrococcus furiosus*), respectively. Further, we report the first application of plastid-based cellulolytic enzymes in the bioconversion of an important industrially pretreated biomass feedstock (*A. donax*) for biofuels production.

## Results

### Vector construction and integration of genes for thermostable cellulolytic enzymes into tobacco plastid genome

**Fig. 1:**
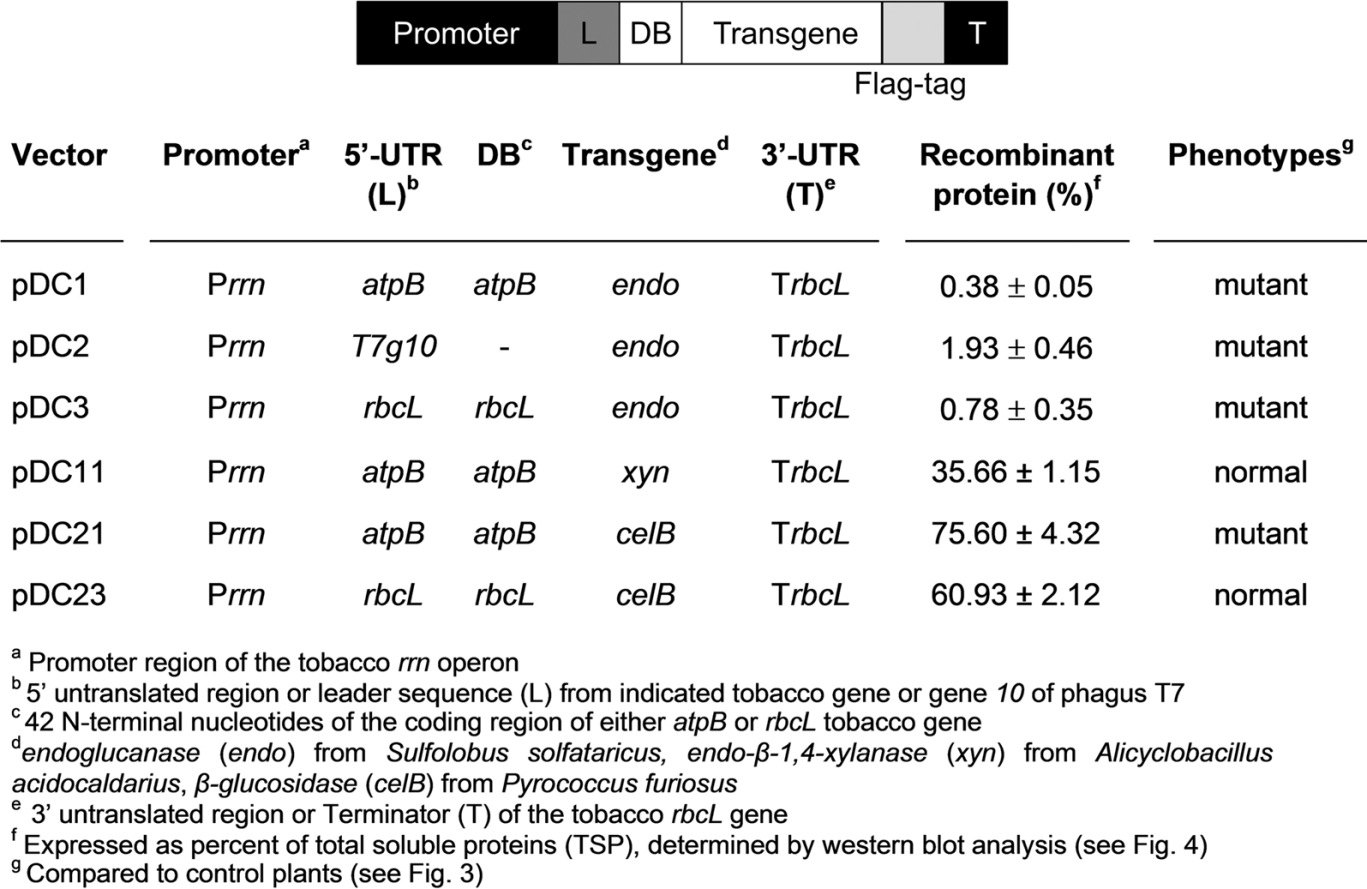
Plastid vectors and expression cassettes used in transformation experiments, containing transgenes encoding cellulolytic enzymes. For each vectors, regulatory sequences, protein accumulation level and phenotype in corresponding transplastomic plants are indicated

**Fig. 2:**
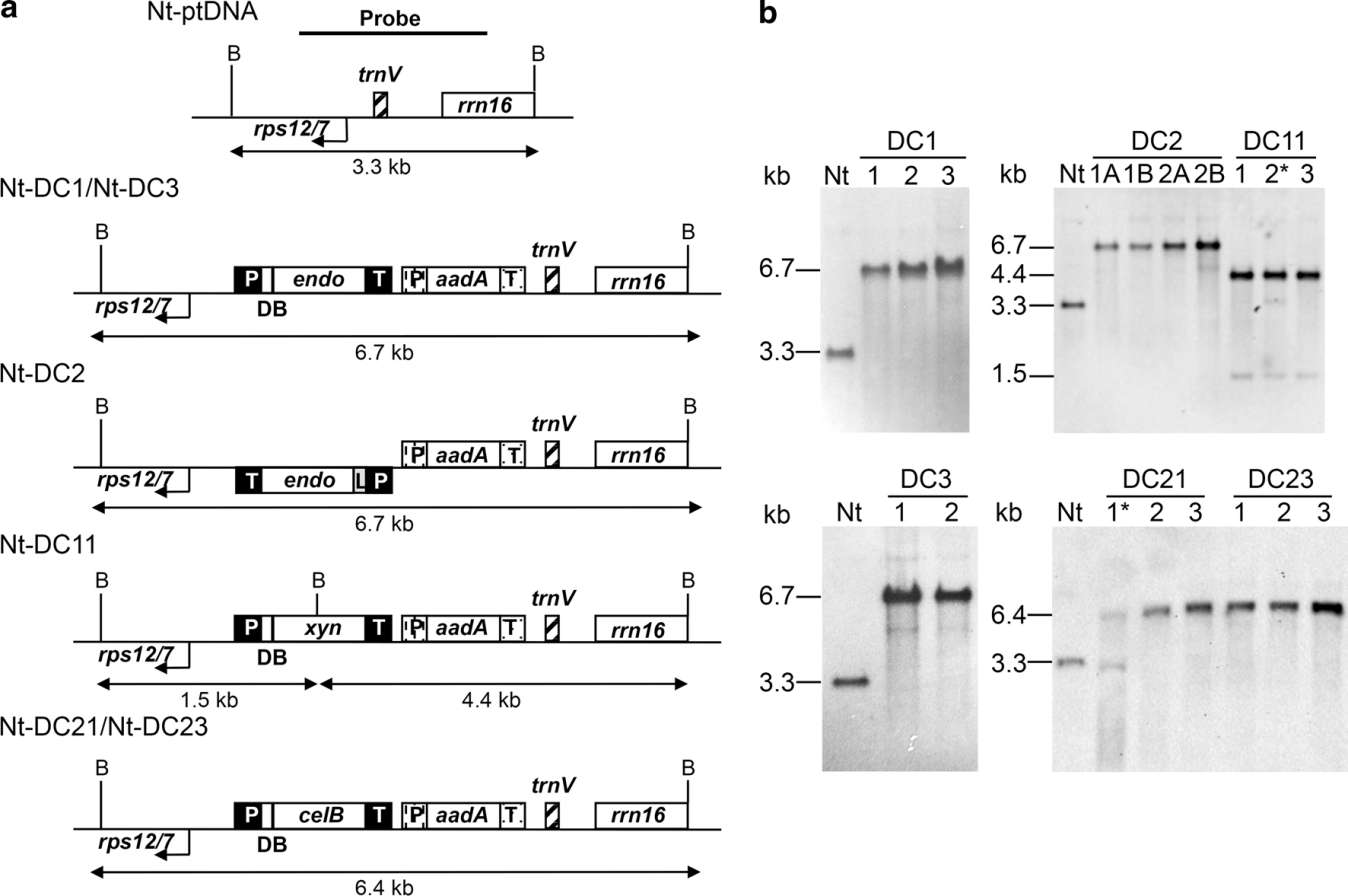
Southern blot analyses of plastid transformants to select independently generated homoplasmic lines per each construct. Schematic representation of the targeting region in the plastid genome (Nt-ptDNA) and maps of transformed (Nt-DC series) plastid genome regions involved in transgene integration (**a**). *Lines* with the same number but *different letters* indicate transplastomic lines generated from the same primary shoot. For DC11 and DC21 transplastomic plants, heteroplasmic lines (marked with an *asterisk*) containing both wild-type and transformed plastomes are also shown (**b**). Below the maps are indicated the expected sizes of the *Bam*HI DNA fragments in Southern blot analyses. *rps12/7* and *trnV* correspond to the plastome integration site of the expression cassettes; *aadA*, *aminoglycoside 3′* *adenylyltransferase* marker gene

To transform tobacco plastid genomes, six vectors containing different subclasses of (hemi)cellulolytic enzymes were developed. In particular, we selected genes from thermophilic or hyperthermophilic bacteria: *endoglucanase* (*endo*) from *Sulfolobus solfataricus*, *endo*-*β*-*1,4*-*xylanase* (*xyn*) from *Alicyclobacillus acidocaldarius*, and *β*-*glucosidase* (*celB*) from *Pyrococcus furiosus.* To ensure high expression level of the transgenes, their coding regions were fused to the strong plastid *rrn* operon promoter in combination with the 5′ untranslated region (5′-UTR) from gene *10* of *E. coli* phage *T7* (*T7g10*) in pDC2, or 5′ translation control region (5′-TCR) that includes the 5′-UTR and 42 N-terminal nucleotides (DB) of the *atpB* (pDC1, pDC11 and pDC21 vectors) or *rbcL* open reading frames (pDC3 and pDC23 vectors). All expression cassettes contain the 3′-UTR of *rbcL* gene. A Flag tag was added at C-terminus of all coding regions for protein detection (Fig. 1). The expression cassettes for transgenes expression were cloned in plastid pPRV vectors [37] targeting exogenous DNA to the *trnV*–*rps12/7* region of the tobacco plastid genome and containing *aminoglycoside 3′adenylyltransferase(aadA)* gene as a selectable marker (Fig. 2).

Plastid transformation experiments, carried out by biolistic method, produced several putative transplastomic lines (about 20 primary spectinomycin-resistant shoots per 10–15 bombardments) with all constructs. To isolate homoplasmic lines, the primary shoots were subjected to one or two regeneration rounds on the medium containing spectinomycin. Spontaneous spectinomycin-resistant mutants (about 35–40 %) were eliminated through a double antibiotic resistance test on the medium containing both spectinomycin and streptomycin. Southern blot analyses, carried out on DNA isolated from 5 to 7 transplastomic plants for each vector and with the targeting sequence as probe, confirmed correct integration and homoplasmy of foreign DNA for all vectors. A 6.7- or 6.4-kb fragment was identified for *endoglucanase* and *β*-*glucosidase* transplastomic plants, respectively; while 4.4 plus 1.5 kb fragments and 3.3 kb fragment for *endo*-*β*-*1,4*-*xylanase* transplastomic and wild-type plants, respectively (Fig. 2).

### Phenotypes of transplastomic plants expressing cellulolytic enzymes

**Fig. 3:**
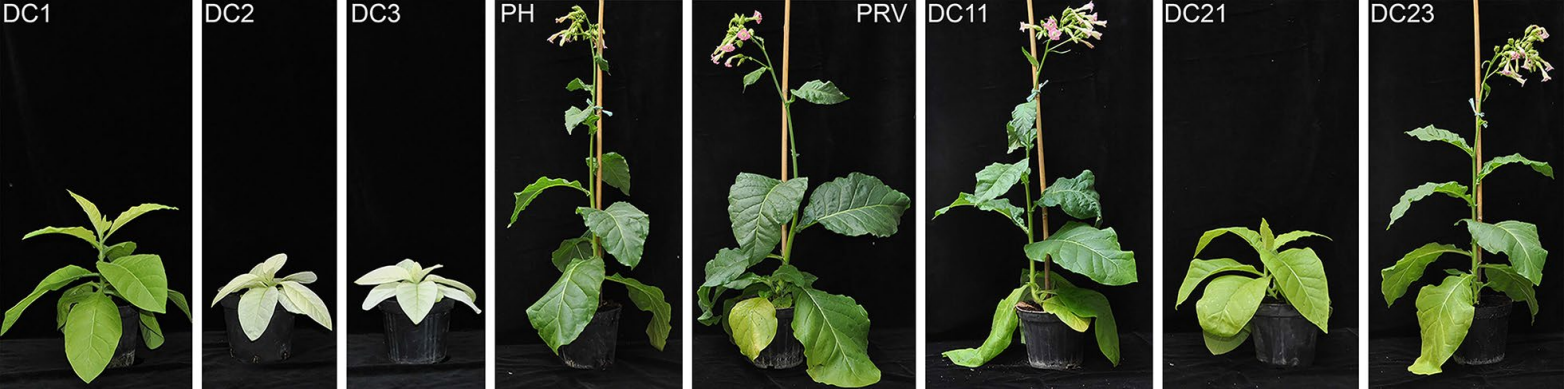
Plant phenotypes. Comparison of plant growth between mature control (PH and PRV, wild-type and transformed with the empty vector, respectively) and transplastomic plants expressing endoglucanase (DC1, DC2 and DC3), endo-β-1,4-xylanase (DC11) and β-glucosidase (DC21 and DC23) cellulolytic enzymes under photoautotrophic conditions in soil

Transplastomic plants expressing cellulolytic enzymes grown in soil under greenhouse conditions showed variable phenotypes compared to control (wild-type and transformed with empty vector) plants (Fig. 3). In particular, the accumulation of the endoglucanase in tobacco chloroplasts produced an altered plant phenotype (pigment-deficient and retarded growth) which severity was directly linked to the protein expression level. However, they were able to reach maturity, flower and produce viable seeds by selfing. Further, all endoglucanase-transplastomic seeds were able to germinate in soil 1 week after sowing, but, in contrast to the control lines, most DC2 seedlings died within 3–4 weeks (Additional file 1: Figure S1). On the contrary, the accumulation of endo-β-1,4-xylanase (DC11) in our transplastomic plants did not interfere with plant growth, in fact these plants exhibited a similar phenotype to control plants either when transferred or germinated in soil (Fig. 3). Similarly to DC11 plants, for transplastomic plants accumulating the β-glucosidase enzyme (DC21 and DC23) no visible phenotypic alteration was observed in young plants, whereas only in DC21 mature plants a severe growth retardation was detected (Fig. 3). Both endo-β-1,4-xylanase and β-glucosidase transplastomic plants produced viable seeds by selfing.

### Expression of cellulolytic enzymes in transplastomic plants

Expression analyses of cellulolytic enzymes were carried out on T1 for endoglucanase and endo-β-1,4-xylanase and T0 transplastomic plants for β-glucosidase, respectively. Northern blot analyses of total RNA from the leaves of pPRV (empty vector), pDC-derived lines were performed using gene-specific probes. For *endoglucanase* gene a transcript of about 2.3 kb was detected in DC2 transplastomic plants, and two transcripts of about 2.3 and 3.6 kb in DC1 and DC3 plants, corresponding to mono- and readthrough dicistronic mRNAs (more abundant), respectively (Additional file 1: Figure S2A); whereas for *endo*-*β*-*1,4*-*xylanase* and *β*-*glucosidase* genes two transcripts of about 1.5 and 2.8, and of about 2 and 3.3 kb were detected in DC11, DC21and DC23 transplastomic plants, respectively (Additional file 1: Figure S2B, C).

**Fig. 4:**
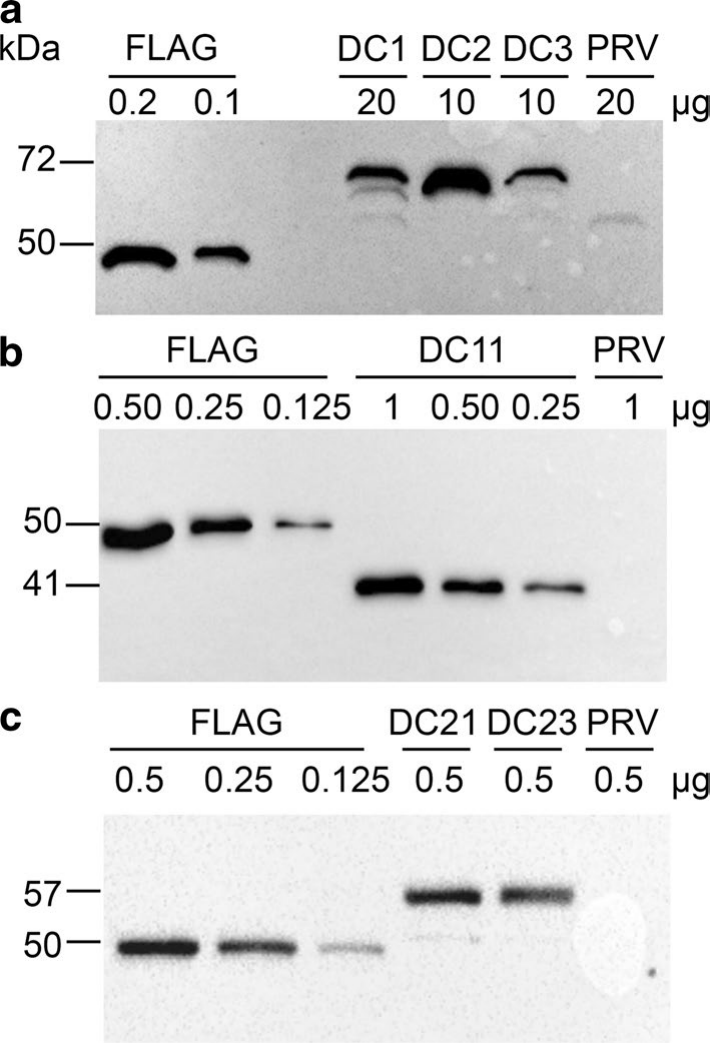
Detection by Western blot analysis of cellulolytic enzymes accumulated in transplastomic DC plants. Each transplastomic line shows a prominent additional protein band corresponding in size to the foreign protein. Endoglucanase (**a**) and endo-β-1,4-xylanase (**b**) enzymes accumulation in DC1, DC2, DC3 and DC11 transplastomic plants, respectively (T1 generation). **c** Accumulation of β-glucosidase enzyme in DC21 and DC23 transplastomic plants (T0 generation). FLAG: recombinant Flag standard protein; PRV: transplastomic control plant transformed with the empty vector

Western blot analysis was carried out with total soluble proteins and monoclonal anti-Flag antibody, and specific proteins of the expected molecular mass were recognized for all enzymes. For endoglucanase protein, we estimated a variable protein yield, 0.38 ± 0.05, 1.93 ± 0.46 and 0.78 ± 0.35 % of total soluble proteins (TSP) in DC1, DC2 and DC3 transplastomic plants, respectively (Fig. 4a). The highest protein yield was obtained with pDC2 vector containing the *T7g10* 5′-UTR regulatory sequence. The endo-β-1,4-xylanase enzyme was accumulated at 35.66 ± 1.15 % TSP (Fig. 4b), whereas the yield of β-glucosidase recombinant protein was 75.6 ± 4.3 and 60.9 ± 2.1 % TSP in pDC21 and pDC23 derived lines, respectively (Fig. 4c).

**Fig. 5:**
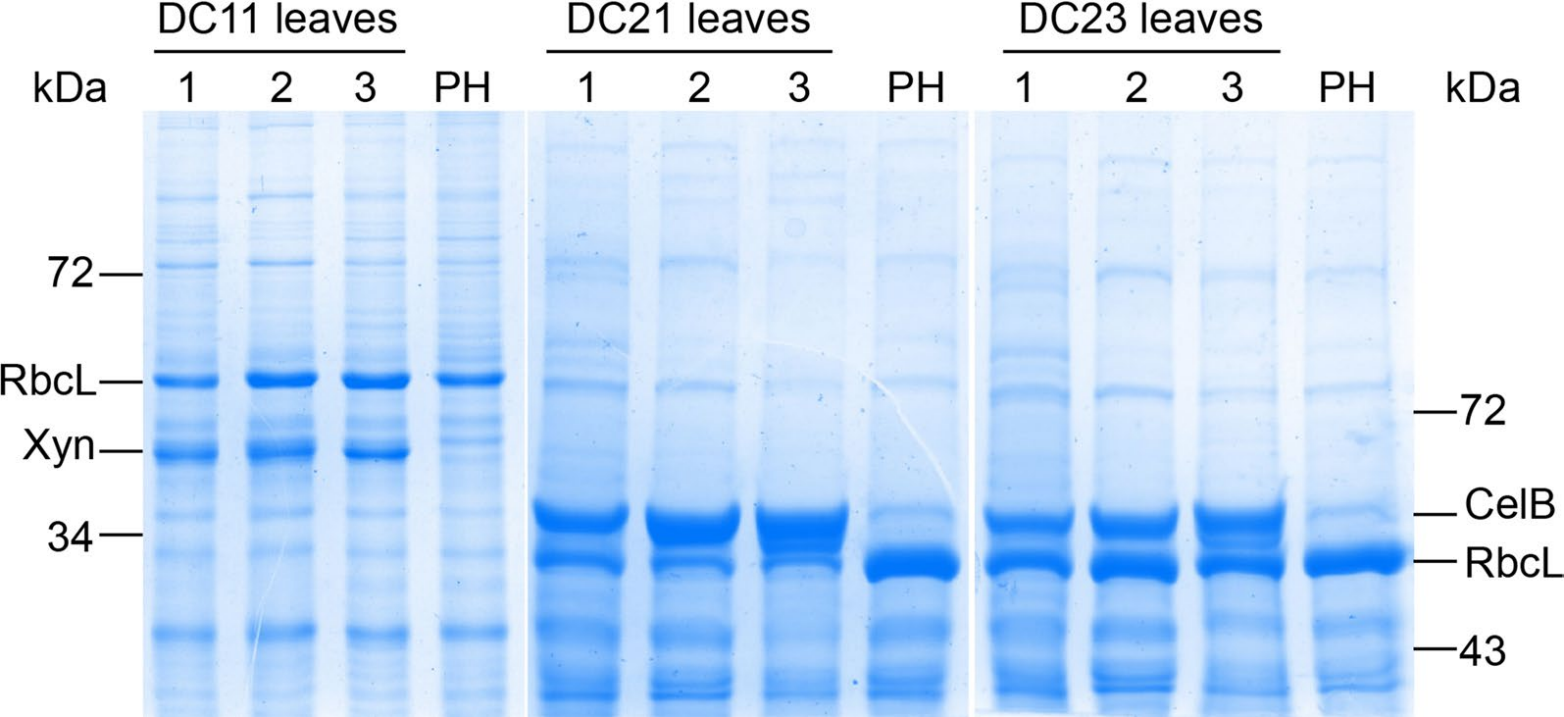
Detection by Coomassie blue staining of polyacrylamide gel of cellulolytic enzymes accumulated in transplastomic DC plants. Each leaf of different age of transplastomic line shows a prominent additional protein band corresponding in size to the foreign protein. *DC11* T1 transplastomic plants expressing endo-β-1,4-xylanase (xyn), *DC21, DC23* T0 transplastomic plants expressing β-glucosidase (celB), *PH* wild-type plant. *1* young leaf; *2* mature leaf; *3* old leaf; *RbcL* Rubisco large subunit protein

Because the protein biosynthesis capacity can decline with leaf age, we also evaluated by Coomassie staining of SDS-PAGE gel, only for the enzymes with the highest yields (endo-β-1,4-xylanase and β-glucosidase), their stability *in planta* analyzing leaves at different development stage (young, mature, old) of autotrophically growing plants. No significant variation in protein accumulation was observed for both endo-β-1,4-xylanase and β-glucosidase enzymes. Recombinant and Rubisco large subunit proteins, the most abundant protein that accumulates to approximately 50–60 % of TSP in plants, accumulated at approximately the same level. Only in DC21 transplastomic plants was a reduction of the endogenous Rubisco large subunit protein (RbcL) observed (Fig. 5).

Northern blot analysis using *rbcL* coding region as probe demonstrated that β-glucosidase-expressing plants accumulated *rbcL* transcripts at the same or higher level than control plants (Additional file 1: Figure S3), suggesting that the reduction of Rubisco large subunit protein was due to an exhaustion of plastid translational capacity in DC21 plants.

Analysis of enzyme yields in T2 (endoglucanase and endo-β-1,4-xylanase) or T1 (β-glucosidase) transplastomic plants, respectively, confirmed stable expression levels also in subsequent generations of transformed plants (Additional file 1: Figure S4).

### Ultrastructural alterations and sub-organellar localization of the endoglucanase

**Fig. 6:**
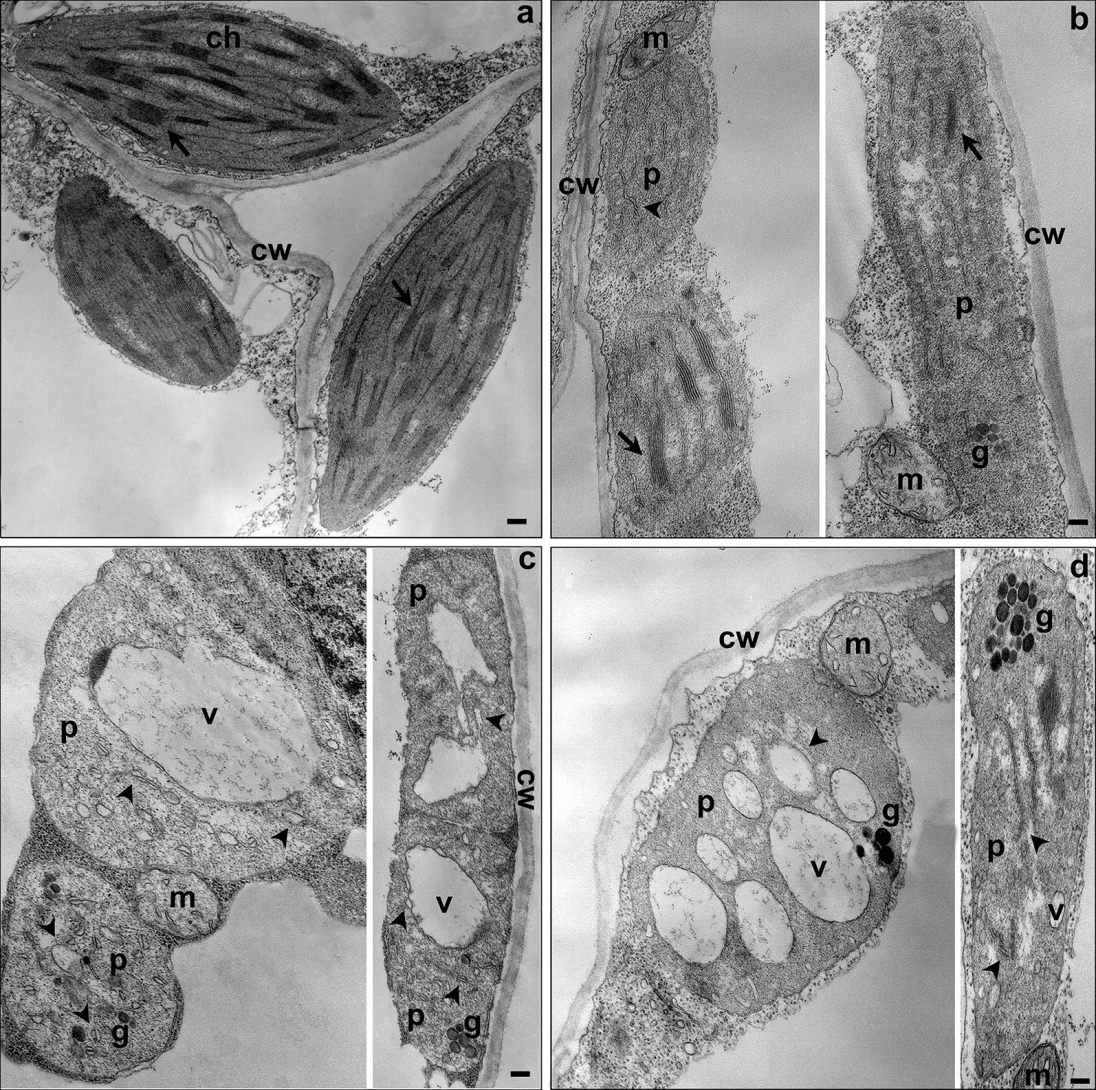
Transmission Electron Microscopy (TEM) images of cells and plastids of control and endoglucanase-expressing transplastomic plants. PRV control plants (**a**) show mature chloroplasts, whereas for endoglucanase-expressing transplastomic plants the coexistence of mature chloroplast and plastid resembling proplastid in DC1 plants (**b**) and the prevalent presence of proplastids containing vesicles in DC2 (**c**) and DC3 (**d**) plants were observed. *Arrows* in **a** and **b** indicate grana and inter-grana structures. *Arrowheads* in **c** and **d** point to rudimentary thylakoids. *Ch* chloroplast, *cw* cell wall, *p* plastid, *m* mitochondria, *g* plastoglobules, *v* vesicles. *Scale bars* 100 nm

To evaluate whether the aberrant phenotype of endoglucanase-expressing plants was linked to the alteration of chloroplasts ultrastructure, Transmission Electron Microscopy (TEM) analysis of mesophyll cells was carried out on transplastomic and control plants. As shown in Fig. 6, transplastomic plants expressing the endoglucanase revealed various changes in chloroplast ultrastructure (Fig. 6b–d), compared to PRV control plants (Fig. 6a) showing mature chloroplasts with grana and inter-grana structures clearly visible (indicated by arrow). In particular, DC1 plants showed the presence of almost mature chloroplasts and a small portion (about 10–20 %) of proplastids at an intermediate development stage containing thylakoid-like membranes (indicated by arrowheads, Fig. 6b); whereas DC2 and DC3 transplastomic plants, characterized by a much more severe plant phenotype than DC1, showed higher percentage of proplastids (80–90 % and 40–50 %, respectively) containing vesicles with electron-dense material of unknown origin (Fig. 6c, d, respectively).

**Fig. 7:**
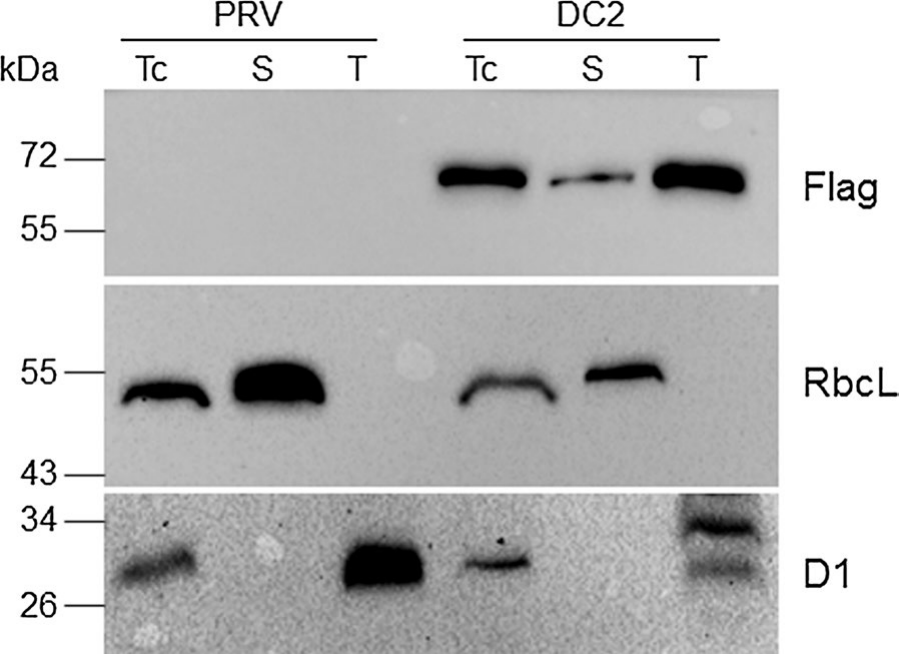
Sub-organellar localization of endoglucanase. Western blot analyses of chloroplast subfractions from control (PRV) and endoglucanase (DC2) transplastomic plants performed using monoclonal and polyclonal antibodies raised against Flag tag, RbcL (stroma marker) and D1 (thylakoid marker). *Tc* total chloroplast proteins, *S* stroma proteins, *T* thylakoid proteins

Further, we also investigated whether the seedling-lethal phenotype of DC2 endoglucanase-expressing plants was also due to the relocation of the foreign protein to the plastidial membranes by Western blot analyses on chloroplast subfractions. Immunoblot analysis with Flag antibodies revealed that the endoglucanase was mostly localized in the thylakoid membranes of the immature chloroplasts (Fig. 7). The purity of each subfraction, and hence the validity of the sub-organellar localization of the endoglucanase, was assessed using specific antibodies corresponding to the stromal RbcL protein and the integral thylakoid membrane protein D1 (core subunit of the photosystem II reaction center) as marker proteins (Fig. 7).

### Biochemical characterization of endo-β-1,4-xylanase and β-glucosidase cellulolytic enzymes produced in tobacco chloroplasts

Based on very high expression level obtained with endo-β-1,4-xylanase and β-glucosidase, we measured the expression level of active enzymes by, standard assays only with these enzymes using 4-*O*-methyl-glucurono-xylan (MGX) and 4-nitro-phenyl-β-d-glucopyranoside (pNPG) as substrates, respectively, and total protein extracts prepared from an old leaf and from a pool of whole plant leaves of DC11 and DC23 transplastomic plants. Between β-glucosidase-expressing plants, due to the phenotypic alteration observed in DC21 plants, DC23 transplastomic plants were selected for enzyme assays. Enzyme activity assays, performed with total protein extracts from whole plant leaves or old leaf of wild-type and transplastomic plants, showed a specific activity of about 27.2 and 34.2 U/mg proteins for endo-β-1,4-xylanase, and 62.6 and 97.2 U/mg proteins for β-glucosidase, respectively (Table 1).

**Table 1:** Enzyme yield in transplastomic tobacco plants and partial purification of plastid-based cellulolytic enzymes

Enzyme	Whole plant leaves (g FW)	% yield (TSP)^a^	Specific activity^b^						Fold purification
			Crude extract					Enriched extracts	
			Old leaf	Whole plants leaves					
			U/mg	U/mg	U/g plant	U/plant	U (millions)/t DW	U/mg	
Endo-β-1,4-xylanase	45.5	35.7	34.2	27.2	163.3	7429.7	1484	105.4	3.9
β-Glucosidase	45.2	60.9	97.2	62.6	1450.8	65,574.6	13,189	255.4	4.1

*g FW* gram of fresh weight leaves, *TSP* total soluble proteins, *U/mg* unit per milligram of crude or enriched extract, *U (millions)/t DW* unit per ton of dry weight leaves

^a^Determined by Western blotting analysis using a dilution series of purified Flag protein

^b^Determined by enzymatic standard assay. Data are relative to crude and enriched extracts by heat treatment (see “Methods” section for details)

**Fig. 8:**
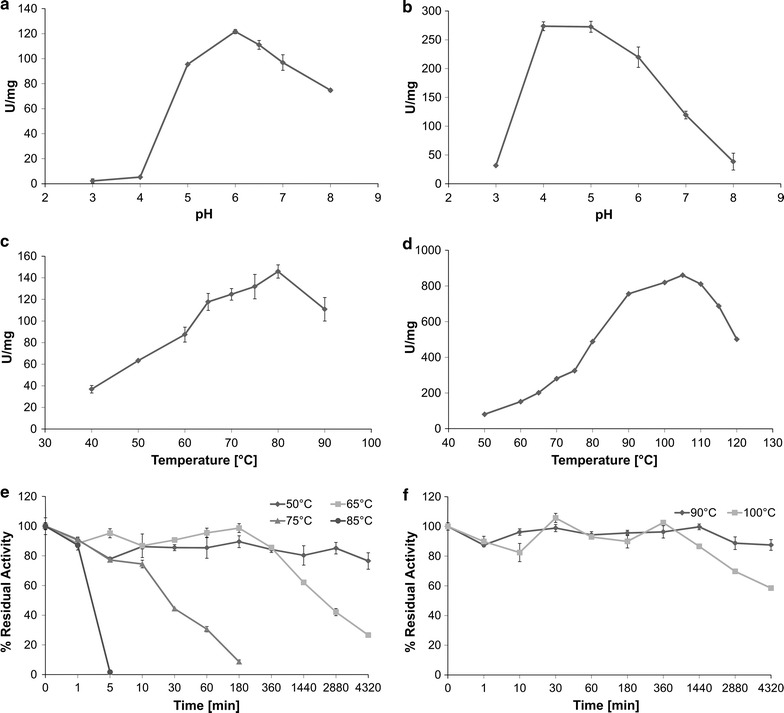
Characterization of cellulolytic enzymes produced in transplastomic plants. Specific activity of endo-β-1,4-xylanase (xyn) vs temperature (**a**) and pH (**c**). Thermostability of xyn at 50, 65, 75 and 85 °C (**e**, *rhombus*, *squares*, *triangles* and *circles*, respectively). Specific activity of β-glucosidase (celB) vs temperature (**b**) and pH (**d**). Thermostability of celB at 90 and 100 °C (**f**, *rhombus* and *squares*, respectively). The assays related to the evaluation of specific activity vs pH were performed in citrate/phosphate and sodium phosphate buffers in the ranges 3.0–6.0 and 6.0–8.0, respectively

Being endo-β-1,4-xylanase and β-glucosidase thermostable enzymes, crude extracts from transplastomic plants were subjected to a simple and low-cost enzyme enrichment based on thermal treatment of crude extracts. In particular, a treatment at 65 and 80 °C for 15 min was applied to DC11 and DC23 crude extracts, respectively. The specific enzyme activities revealed an enrichment of about fourfold (Table 1) for both enzymes (105.4 and 255.4 U/mg proteins for endo-β-1,4-xylanase and β-glucosidase, respectively); hence, the enriched extracts were used for enzymes characterization. Similar specific enzyme activities were also obtained in crude and enriched extracts from T2 and T1 generations of DC11 and DC23 transplastomic plants, respectively (Additional file 1: Table S1). Total protein extracts from pPRV transplastomic or wild-type plants were used as negative controls in all standard assays and revealed no specific enzyme activities. The endo-β-1,4-xylanase (xyn) showed its maximum at 80 °C and high activity levels were also detected between 60 and 90 °C, with values of about 60 and 80 % of the optimum at the extremes of this temperature range (Fig. 8a). The activity assay at different pH revealed for our endo-β-1,4-xylanase an optimal pH 6.0 in sodium citrate and sodium phosphate buffers at 65 °C and that the enzyme was active over the whole pH range 4.0–8.0 (Fig. 8c). Thermostability experiments demonstrated that plastid-based xyn was quite stable being still active after 3 days of incubation at 50 °C and maintaining 65 % specific activity after 24 h of incubation at 65 °C (Fig. 8e).

The β-glucosidase (celB) expressed in tobacco plastome exhibited its optimal activity at 105 °C and pH 4.0–5.0, and worked over a broad range of high temperatures (80–120 °C) retaining over 60 % of its maximal activity (Fig. 8b, d). In these conditions, celB showed also a remarkable thermostability with residual activities of 60 and 90 % after 72 h at 100 and 90 °C, respectively (Fig. 8f).

### Enzymatic hydrolysis of an industrially pretreated *Arundo donax* biomass

To evaluate the enzymatic potential of the recombinant endo-β-1,4-xylanase and β-glucosidase produced in tobacco chloroplasts to promote the saccharification of lignocellulosic feedstock, these biocatalysts were used as components of an enzymatic cocktail applied to *A. donax* biomass industrially pretreated by steam explosion [38].

The macromolecular composition of the pretreated material in terms of percentage of glucans, xylans and Klason lignin is 38.2, 5.7 and 36.1, respectively.

The adopted strategy was to determine the kinetic yields of glucose and xylose released up to 72-h incubation of the pretreated biomass in the presence of cellulose-degrading enzymes (cellulase and β-glucosidase) and xylan-related activities (xylanase and β-xylosidase). Cellulose and xylan conversion, when conducted for 72 h at 50 °C in the presence of the commercial (hemi)cellulolytic activities (see Mix 1 composition under “Methods” section and Table 2), gave glucose and xylose yields of 51.98 and 98.86 %, respectively. The replacement of the xylanase from *T. viride* in the Mix 1 with the plastid-based xyn (Mix 2) allowed to reach almost the same xylose yield obtained after 72 h with the commercial counterpart (Table 2). In addition, the xylan hydrolysis was speeded up as witnessed by an increase of 7.56 % for the xylose recovery after 48 h when reached about 87 %. The glucose yield underwent a 32 % reduction after 48 h of incubation leading to a monosaccharide recovery of 30.5 %. The replacement of both the commercial xylanase and β-glucosidase with the in house plastid-developed enzymes (Mix 4) did not had positive effects on glucose recovery and slowed down the rate of xylan breakdown by 6 % after 72 h of incubation (93 vs 99 % yields) (Table 2). The impact of protein loading on the hydrolysis efficiency was also evaluated, but using a twofold protein loading did not caused additional benefits to cellulose and xylan conversion (data not shown).

**Table 2:** Biotranformations of industrially pretreated *A. donax* biomass in the presence of commercial and plastid-based cellulose degrading enzymes

Composition	Glucose yield (%)			Xylose yield (%)		
	24 h	48 h	72 h	24 h	48 h	72 h
50 °C						
Mix 1	36.84 ± 1.10	44.94 ± 0.10	51.98 ± 0.60	77.51 ± 2.90	80.66 ± 0.80	98.86 ± 3.60
Mix 2	27.20 ± 1.30	30.46 ± 0.90	41.15 ± 1.70	72.22 ± 2.10	86.76 ± 3.70	95.84 ± 4.90
Mix 3	19.07 ± 1.36	27.39 ± 1.62	34.21 ± 0.97	60.65 ± 2.40	74.89 ± 2.73	90.99 ± 2.91
Mix 4	17.99 ± 0.10	26.99 ± 0.90	32.99 ± 0.10	52.97 ± 1.90	71.72 ± 0.80	92.74 ± 1.00
60 °C						
Mix 1	14.99 ± 0.46	18.93 ± 1.32	20.00 ± 0.06	45.67 ± 0.16	55.25 ± 2.36	60.53 ± 0.37
Mix 2	18.20 ± 1.17	21.43 ± 1.40	23.05 ± 0.13	56.09 ± 3.81	66.70 ± 0.98	72.61 ± 1.22
Mix 3	15.72 ± 0.60	18.59 ± 0.91	22.32 ± 1.13	43.26 ± 1.82	53.16 ± 2.43	63.17 ± 2.14
Mix 4	15.23 ± 0.67	18.8 ± 0.96	20.12 ± 0.77	53.27 ± 2.09	61.83 ± 2.09	70.15 ± 1.21

Mix 1: commercial mix composed by cellulase from *Trichoderma reesei* ATCC 26921 (5.4 U/g of pretreated biomass); β-glucosidase from *Aspergillus niger* (145 U/g); xylanase from *Trichoderma viride* (80 U/g); β-xylosidase (8 U/g)

Mix 2: Mix 1, in which plastid-based xylanase (xyn) from *Alicyclobacillus acidocaldarius*, (80 U/g) replaced commercial xylanase from *T. viride*

Mix 3: Mix 1, in which plastid-based β-glucosidase (celB) from *Pyrococcus furiosus* (145 U/g) replaced β-glucosidase from *A. niger*

Mix 4: Mix 1, in which plastid-based xylanase (xyn) from *A. acidocaldarius*, (80 U/g) and β-glucosidase (celB) from *P. furiosus* (145 U/g) replaced xylanase from *T. viride* and β-glucosidase from *A. niger,* respectively

At 60 °C the maximum xylose recovery with Mix 1 (60.53 % after 72 h) was increased by 20 % after the substitution of the commercial xylanase with the in plant expressed enzyme (Mix 2, 72.6 %). The xylanase replacement, besides, speeded up the xylan degradation (23 and 21 % increase in xylose recovery after 24 and 48 h, respectively). Differently from the results obtained at 50 °C, when also the β-glucosidase from *A. niger* was replaced by the plastid-derived celB (Mix 4), the glucose yield was almost identical, whereas the xylose recovery and hydrolysis rates were boosted. The replacement of the commercial biocatalyst with β-glucosidase from *P. furiosus* expressed in tobacco plastome (Mix 3) at 50 °C reduced both the glucose and xylose recovery, while at 60 °C maintained the high monosaccharides yield (Table 2).

## Discussion

Over past decades, it has been demonstrated that many genes can be expressed at high levels when introduced in the plastid genome [39–44]. Although the high copy number of the plastid genome per cell is generally assumed to be responsible for high foreign protein yields, other factors, such as expression signals (promoters, 5′-UTR or -TCR, 3′-UTR), the codon usage or plant tissue and species, could affect the transgene accumulation by plastid transformation [45–47].

In this work, we explored the possibility to increase the (hemi)cellulolytic proteins accumulation in transgenic plastids and employed plants as cheap production platform of thermophilic and hyperthermophilic enzymes for second-generation biofuels. The choice of (hemi)cellulolytic enzymes isolated from thermophilic and hyperthermophilic microorganisms was based on the necessity to develop bioconversion process at high temperature (>50 °C) to obtain a faster enzymatic hydrolysis rate and higher yields of sugars. All selected enzymes were expressed in transgenic chloroplasts at variable levels. We estimated a protein yield range from 2 % to more than 75 % of total soluble proteins for hyperthermophilic endoglucanase and β-glucosidase isolated from *Archaea* (*S. solfataricus* and *P. furiosus*, respectively). The high expression level of β-glucosidase, obtained using 5′-TCRs as regulatory sequences, did not cause altered phenotypes, except in DC21 transplastomic plants that show a growth retardation only in mature plants grown in soil. This phenotype was likely due to the extremely high expression level (>75 % TSP) of β-glucosidase and a concomitant reduction of the most abundant plant protein, Rubisco. Similarly to what observed for the synthetic phage-derived bactericidal lysin protein [29], the reduction at protein level of Rubisco in DC21 transplastomic plants was not accompanied by a reduction of *rbcL* transcripts, suggesting an exhaustion of the protein synthesis capacity due to massive expression of β-glucosidase. This hypothesis was also confirmed by the fact that other transplastomic plants (DC23) expressing β-glucosidase at lower level (≥60 % TSP) did not show neither growth retardation nor reduction of Rubisco protein. The β-glucosidase yield produced in this work represents the highest accumulation level of cellulolytic enzymes produced in plants so far, and in particular, for a hyperthermophilic archaeal enzyme. On the contrary, the accumulation of another hyperthermophilic enzyme in tobacco transplastomic plants, an uncharacterized endoglucanase, belonging to the family 5 of glycoside hydrolase, from *S. solfataricus*, gave a maximum yield of about 2 % TSP (DC2 plants) using the 5′-UTR of gene *10* of the phage T7 as regulatory sequence, and a marked alteration of plant phenotypes whose severity was directly linked to the enzyme expression level. Seedling growth in soil revealed, within 3–4 weeks after germination, a seedling-lethal phenotype in DC2 transplastomic plants, with seedling development being blocked at the cotyledon stage, whereas for other endoglucanase-expressing plants (DC1 and DC3) seedlings were able to develop in plants with different pigment-deficiency and growth retardation alterations. As previously reported, the high expression level of recombinant proteins cannot be the only cause of mutant phenotype [29, 47–51]. In fact, Petersen and Bock [23] tentatively attribute the severity of the phenotypes of their transplastomic plants, expressing cellulolytic enzymes in a range of 5–40 % TSP (endoglucanase up to 40 %), to the carbohydrate-binding activity of recombinant enzymes through sequestration or degradation of intermediates in plant carbohydrate metabolism. By contrast, the most severe phenotype observed in our DC2 transplastomic plants was due to the impairment of plastid development associated to the binding of endoglucanase protein to thylakoids, as demonstrated by electron microscopy and immune blot analyses. In particular, they showed chloroplasts with irregular shape and a severely defective ultrastructure characterized by rudimentary and unstacked internal membranes dispersed in an electron-dense matrix, resembling to proplastids. Similar results were also observed in our laboratory for tobacco transplastomic plants expressing HIV-1/Pr55^gag^ [52].

Accordingly to lower endoglucanase accumulation levels, DC1 and DC3 transplastomic plants were characterized by an alteration of plastid ultrastructure and plant phenotype less severe than DC2 plants.

As reported above for β-glucosidase, the high expression level up to 36 % TSP of thermophilic endo-β-1,4-xylanase from *A. acidocaldarius* was obtained using the 5′-TCR of *atpB* gene. Although our xylanase yield was the highest reported until now by plastid transformation [18, 19, 33, 35], transplastomic plants did not show pleiotropic effects, whereas Kolotilin et al. [19] observed variable phenotypic alterations in transplastomic plants expressing xylanase under the control of different regulatory sequences and directly linked to the protein expression level.

As clearly demonstrated in this and other works, the expression of cellulolytic enzymes in the chloroplast genome, when the coding sequences does not contain thylakoid- or carbohydrate-binding motifs, enables, through the proteins containment in the organelle, higher accumulation level and stability by reduced exposure to protease, and keeps the cell wall-degrading enzymes away from cytoplasmic metabolic activities and host cell walls, avoiding potential damage to plant host.

The enriched extracts obtained by heat treatments were characterized to obtain the operational parameters necessary for the enzymes application in the process of lignocellulosic biomasses saccharification. Both the biocatalysts expressed in transplastomic tobacco plants substantially retained the main features of the native (celB) or recombinantly expressed enzymes (xyn) [53, 54]. These findings are consequence of the strict similarity between the prokaryotic and plastidial genetic systems that is supported by the endosymbiotic theory for the organelle origin [42]. However, some interesting differences have to be pointed out. As concerning the effect of the temperatures on the xylanase activity, the protein expressed in tobacco plastome has proved to be more thermophilic than the *E. coli* recombinant counterpart. In fact, the maximal activity was reached at 80 °C instead of 75 °C and above the temperature optimum the activity decrease was less pronounced than that evidenced for the recombinant protein expressed in the bacterial host (80 % instead of 65 % residual activity at 85 °C). Moreover, xyn activity expressed in transplastomic tobacco remained high at acidic pH (80 and 50 % at pH 5.0 and 4.5, respectively) differently from the *E. coli*-derived enzyme. Also the plastid-based β-glucosidase revealed a pronounced thermophilicity. As a matter of fact, differently from the native protein, whose activity rapidly decreased above 105 °C, the enzyme retained 90 and 66 % of the maximal activity at 115 and 120 °C, respectively. On the other hand, the activity of the enzyme drops sharply as the temperature declines, displaying only 23 % activity at 65 °C and very low values below 50 °C. These findings suggest that there would be no hydrolysis of glycosidic bonds under normal plant growth conditions and support the absence of phenotypic alteration observed in DC23 transplastomic plants.

The ultimate goal of the (hemi)cellulolytic enzymes production in plants is their utilization in cellulosic biomass conversion. The exploitation of crops that are not employed for nutritional uses such as switchgrass, *Arundo donax* and *Miscanthus* match the principal objectives of the second-generation biofuels, increasing their sustainable production [55]. Among them, *A. donax* is considered as one of the ‘energy crops’ with highest potentialities owing to its high (hemi)cellulose content, fast growth rate, high biomass yields also on marginal soils and at different climatic conditions [1, 56, 57].

To improve the (hemi)cellulose digestibility the raw materials must be subjected to a pretreatment that remove the recalcitrant lignin fraction which shields the cell wall polysaccharides from the enzymatic attack. Previous papers dealt with bioconversions of pretreated biomasses [58–60] through the utilization of thermophilic enzymes produced in transgenic plants. The present paper represents the first report on the application of plastid-derived (hemi)cellulolytic enzymes for the bioconversion of an industrially pretreated, by steam explosion, giant reed biomass.

As a comparison, we used commercial enzymes, namely a cellulase from *T. reesei*, a β-glucosidase from *A. niger*, a xylanase from *T. viride*, and a β-xylosidase. These enzymes were used as controls and as mesophilic model counterparts of the enzymes under study here, in fact, enzymatic cocktails used in production plants for lignocellulose degradation include tens of different activities optimized for the operational conditions.

It is worth noting that, when the plant recombinant xyn replaced the commercial xylanase (Mix2) at 50 °C, the recovery of xylose remains similarly high and the xylan hydrolysis was even speeded up. The slightly lower glucose recovery observed with Mix2 suggests a synergistic action between the xylanase from *T. viride* and the cellulase from *T. reesei* in the degradation of the biomass that was less efficient when the former was replaced by the plastid-derived xyn. The glucose recovery was not increased after the replacement of the commercial β-glucosidase with plastid-derived celB, because at this temperature the enzyme activity was very poor. This had a negative impact on the xylan degradation too [61]. Due to the thermophilic nature of the plastid-derived enzymes, the experiments were carried out also at higher temperature (60 °C). After the replacement of the commercial xylanase with plastid-derived xyn, positive results in terms of both xylose recovery and hydrolysis rate were obtained. Upon the substitution of both the xylanase and glucosidase, it is worth noting that glucose levels remained as high as those observed with the commercial cocktail thereby improving the yields observed at 50 °C. Instead, remarkably, xylose yields were always higher in the whole 24–72 h range. It is worth mentioning that these results were obtained even at a temperature possibly hampering the activity of the commercial cellulase that might have thereby limited the amount of substrate available to the highly thermophilic celB.

The exploitation of two characterized plastid-derived biocatalysts gave the advantage to evaluate the role of the individual enzymes in the biomass hydrolysis to obtain an optimal enzyme cocktail [62]. In fact, it was possible to demonstrate that plastid-derived xyn produced an increase of the enzymatic hydrolysis rate of sugars and xylose yield when the biomass conversion was carried out at higher temperature (60 °C) than conventional procedure.

## Conclusions

The very high production level of thermophilic and hyperthermophilic enzymes, their stability and bioconversion efficiencies described in this study demonstrate that plastid transformation represents a real cost-effective production platform for cellulolytic enzymes. Despite successful and stable production of plastid-based cellulolytic enzymes have been proved in all plant generations tested, and based on thermophilicity of these recombinant biocatalysts, further work is necessary for an in-depth evaluation of their performances in biomass conversion. In fact, it is worth mentioning that no efforts were made here to further optimize the already good conversion efficiencies of the (hyper)thermophilic enzymes. Better results would be achieved by the formulation of new cocktails combining plastid-derived enzymes produced in this study with commercially available biocatalysts characterized by more similar biochemical properties.

## Methods

### Plant material and growth conditions

Plants of *Nicotiana tabacum* L. *cv*. Petite Havana for plastid transformation were grown in sterile culture conditions on growth regulator-free medium containing MS salts and B5 vitamins (Duchefa, The Netherlands), 30 g/l sucrose and 8 g/l agar, pH 5.6, at 24 ± 2 °C with a 16 h photoperiod of 40 µmol photons m^−2^ s^−1^. Seeds derived from transplastomic plants transformed with genes encoding cellulolytic enzymes (DC series) or with the empty control vector (PRV) were sown in vitro under controlled conditions (16 h light 40 µmol photons m^−2^ s^−1^ and 8 h dark at 24 °C) on Murashige & Skoog (MS) medium with B5 vitamins (Duchefa), solidified with 0.8 % (w/v) agar, with 20 g/l sucrose and 500 mg/l of spectinomycin, or they were sown in soil in a growth chamber (14 h light, 200 µmol photons m^−2^ s^−1^, at 25 °C, and 10 h dark at 20 °C).

### Construction of chloroplast transformation vectors

All pDC vectors were constructed by replacing the *neo* coding gene in plasmids pHK30 and pHK40 [63] or the *L1* coding region in plasmid pPL66 [64]. All plasmids were linearized with *Nhe*I and *Xba*I restriction enzymes. The coding region of the SSO3007 *endoglucanase* (GenBank accession number SSO_RS14590) was PCR-amplified to introduce *Nhe*I sites at both 5′- and 3′-termini and Flag tag at 3′-terminus and cloned in pHK30, pHK40 and pPL66 to generate pDC1, pDC2 and pDC3, respectively. The *endo*-*β*-*1,4*-*xylanase* (GenBank accession number KJ466334) fused with a 3′ Flag tag was cloned, as *Nhe*I–*Xba*I fragment, in pHK30 plasmid to produce pDC11 vector; whereas the *β*-*glucosidase* (GenBank accession number AAC25555.1) fused with a 3′ Flag tag, as *Nhe*I–*Xba*I fragment, was inserted in pHK30 and pPL66 plasmids. The expression cassettes of pDC1, pDC11 and pDC21 vectors contain the *rrn* promoter fused with the 5′ translation control region (5′-TCR), that includes the 5′-UTR and 42 N-terminal nucleotides of the *atpB* open reading frame, and the plastid *rbcL* gene 3′-UTR, those of pDC3 and pDC23 contain the *rrn* promoter fused with the 5′-TCR of the *rbcL* open reading frame, and the plastid *rbcL* gene 3′-UTR, whereas pDC2 expression cassette includes the *rrn* promoter, the 5′-UTR of *E.coli* phage T7 gene *10* and the plastid *rbcL* gene 3′-UTR.

### Stable plastid transformation

DNA for plastid transformation was prepared using the Plasmid Maxi Kit (Qiagen, Germany). *Nicotiana tabacum* leaves were bombarded with gold particles (0.6 μm) coated with pDC vectors DNA, using the Bio-Rad (California, USA) PDS-1000/He Biolistic gun.

Spectinomycin-resistant calli and shoots were selected on RMOP medium containing 500 mg/l spectinomycin dihydrochloride [37]. Resistant shoots were tested on a medium containing both spectinomycin and streptomycin (500 mg/l each), and the positive shoots were taken through one round of regeneration on the selective media with only the former antibiotic. Plants were rooted on medium containing 3 % (*w/v*) sucrose, MS salts, B5 vitamins, 0.8 % (*w/v*) agar and 500 mg/l of spectinomycin in sterile culture conditions.

### Southern blot analysis

Total DNA was isolated from leaves of in vitro grown transformed and untransformed plants (100 mg) with the DNeasy^®^ Plant Mini kit (Qiagen). Leaf DNA (1–3 μg) of transplastomic and control plants was digested with *Bam*HI, electrophoresed on 0.8 % agarose gel and transferred to a nylon membrane (Hybond N^+^; GE Healthcare, Wisconsin, USA) under alkaline conditions (0.4 N NaOH). *rrn16*–*rps12* plastid region, labeled with Digoxigenin-11 dUTP (Roche Applied Science, Germany), was generated by PCR. Hybridizations were carried out at 42 °C following the DIG High Prime DNA labeling and Detection starter kit II manufacturer’s instructions (Roche Applied Science). Chemiluminescent signal was measured using a ChemiDoc™ XRS+ and images analyzed using the Image Lab™ Software (Bio-Rad).

### Northern blot analysis

Total RNA was extracted from leaf tissue of PRV and DC transplastomic plants according to the protocol described on the TIGR website (http://www.tigr.org/tdb/potato/images/SGED_SOP_3.2.1.pdf) and precipitated with LiCl. For northern blot analyses, 5 µg of total RNA for each sample were separated by electrophoresis in 1.2 % agarose/formaldehyde gels and blotted onto Hybond N^+^ membranes (GE Healthcare). Gene-specific probes, labeled with Digoxigenin-11 dUTP (Roche Applied Science), were generated by PCR. Hybridizations were carried out at 42 °C following the DIG High Prime DNA labeling and Detection starter kit II manufacturer’s instructions (Roche Applied Science). Chemiluminescent signal was measured as described for Southern blot analysis.

### Protein extraction and immunoblot analyses

Total soluble protein was extracted from leaf samples homogenized in a buffer containing 50 mM HEPES–KOH (pH 7.5), 10 mM KCH_3_CO_2_, 5 mM Mg(CH_3_CO_2_)_2_, 1 mM EDTA, 1 mM DTT, 1 mM PMSF, 1× Complete proteinase inhibitor (Sigma, Missouri, USA) and 1 % β-mercaptoethanol. The homogenate was centrifuged to remove the insoluble material. Crude protein extracts of DC11 and DC23 transplastomic plants were subjected to thermal treatment at 65 and 80 °C for 15 min, respectively, and subsequently centrifuged at 16,000×*g* for 30 min at 4 °C to remove insoluble proteins. Protein concentrations were determined with a Bio-Rad Protein Assay reagent using bovine serum albumine as a standard. Protein extracts were boiled for 5 min in loading buffer (65 mM Tris–HCl pH 6.8, 2 % SDS, 10 % glycerol, 5 mM DTT, 2 mM EDTA, 0.1 % bromophenol blue) and electrophoresed in a 10 or 12 % SDS-PAGE. Gels were either stained with Coomassie Brilliant Blue R-250 (Sigma) or blotted onto nitrocellulose membrane (Hybond ECL, GE Healthcare). Recombinant FLAG protein (cod. P7582, Sigma) was used as controls. After blocking in T-PBS (Tween 20 0.05 %- PBS)—5 % milk, the blots were incubated with a 1:4000 dilution of monoclonal mouse anti-Flag M2 antisera (Cod. F3165, Sigma). A HRP-conjugated anti-mouse antibody (1:80,000 dilution, cod. A9044, Sigma) was applied for 1 h at room temperature. An ECL plus chemiluminescence reagent (GE Healthcare) was used for detection. The expression levels were quantified on triplicates using a dilution series of purified Flag protein and Bio-Rad Image Lab software.

### Chloroplast purification and protein extraction

Chloroplasts were isolated from leaves of PRV and DC2 transplastomic plants by purification on Percoll gradients [65]. For Western blot analysis, intact chloroplasts were lysed and sub-fractionated by sucrose gradient centrifugation according to the procedures reported by Salvi et al. [65]. Proteins from intact chloroplasts, stroma and thylakoid subfractions were electrophoresed in a 10 or 12 % SDS-PAGE and blotted onto nitrocellulose membranes (Hybond ECL, GE Healthcare). Antisera used in the immunoblot assay were anti-Flag (Cod. F3165, Sigma; diluted 1:4000), anti-RbcL (AS03037, Agrisera, Sweden, diluted 1:10,000) and anti-D1 (AS06124A, Agrisera, diluted 1:1000). Detection and chemiluminescence measurements were carried out as described in “Protein extraction and immunoblot analyses” section.

### Transmission electron microscopy

For thin sectioning, leaf pieces from expanded leaves of PRV and DC1, DC2, DC3 transplastomic plants were processed according to standard procedures [66] and observed with a Philip Morgagni 282D electron microscope.

### Standard assays

Standard assays were performed using crude extracts or an enrichment of plastid-based (hemi)cellulolytic enzymes obtained by thermal treatments of crude extracts (‘enriched extracts’). The standard assay for endo-β-1,4-xylanase was carried out on 5 mg/ml of 4-*O*-methyl-glucurono-d-xylan (MGX) in 50 mM sodium phosphate buffer at pH 6.5 and 65 °C using 1 µg of plastid-based crude or enriched extracts in the final volume of 0.1 ml. The relative activity was measured by Somogyi–Nelson assay as also reported by Cobucci-Ponzano et al. [53], considering the amount of reducing sugar released after 1 min. One unit (U) of enzyme activity was defined as the amount of enzyme releasing 1 µmol of reducing equivalents per minute at the conditions described.

For β-glucosidase, standard assay was performed on 2 mg/ml of 4-nitrophenyl β-d-glucopyranoside (pNPG) in 50 mM sodium citrate buffer at pH 5.4 and 65 °C using 0.25 µg of plastid-based crude or enriched extracts in the final volume of 0.5 ml. The reaction was stopped by adding 1 ml of 1 M Na_2_CO_3_. The relative activity was measured spectrophotometrically at 405 nm and enzyme activity was calculated using the molar extinction coefficient for pNPG (ε 14.7 mM^−1^ cm^−1^). One unit (U) of enzyme activity was defined as amount of enzyme required to liberate 1 µmol of *para*-nitrophenol per minute at standard conditions described. In both cases, the mix of assay was pre-incubated at the indicated temperature for 2 min before adding the enzyme.

### Thermostability, temperature and pH effect

The endo-β-1,4-xylanase thermostability was evaluated by incubating ‘enriched extract’ (2 µg/ul) in PBS buffer pH 7.4 at 50–85 °C temperature range; at appropriate time intervals, aliquots were withdrawn and assayed at standard conditions.

For β-glucosidase thermostability, 6 µg/ml of ‘enriched extract’ was incubated in 200 mM Tris buffer pH 8.0 at 90–100 °C; at appropriate time intervals, aliquots were withdrawn and assayed at standard conditions.

The optimal temperature was measured by assaying enzyme activity at different temperatures in the range of 40–90 °C and 50–120 °C for endo-β-1,4-xylanase and β-glucosidase enzymes, respectively, at standard conditions.

The effect of pH on endo-β-1,4-xylanase and β-glucosidase activities was carried out by assaying ‘enriched extract’ at standard condition using sodium citrate and sodium phosphate buffer in the range 3.0–6.0 and 6.0–9.0, respectively.

### Saccharification of pretreated *A. donax*

The biomass of *A. donax*, pretreated by steam explosion [38] and kindly provided by BioChemtex, was subjected to enzymatic hydrolysis experiments, carried out for 72 h in a total volume of 2.5 ml (50 mM sodium phosphate buffer pH 6.0 plus enzymatic cocktail) at a solid loading of 5 %. The enzyme cocktails (Mix 1) were set up with the following commercial preparations, obtained from Sigma, at the indicated units per gram of pretreated biomass: 5.4 U/g Cellulase from *Trichoderma reesei* ATCC26921, 145 U/g of Cellobiase from *Aspergillus niger*, 80 U/g of Xylanase from *T. viride*, 8 U/g of β-xylosidase. Moreover, two plastid-based biocatalysts, expressed in *N. tabacum*, replaced the commercial counterparts: the xylanase (xyn) and the β-glucosidase (celB) activity from *A. acidocaldarius* and *P. furiosus*, respectively.

The saccharification mixtures together with blanks (pretreated lignocellulosic material without enzyme cocktail) were incubated in a shaking ThermoMixer C (Eppendorf) at 600 rpm, for 72 h and two different temperatures (50 and 60 °C) were tested. Samples withdrawn at different time intervals, cooled on ice, were centrifuged at 16,500×*g* for 30 min at 4 °C and the amount of sugars released were quantified in the collected supernatants, as described below. The saccharification yields, expressed as percentages respect to the sugars content of the pretreated materials before the hydrolysis, are means of three replicates.

### Analytical method for sugars determination

The sugars released after enzymatic hydrolysis were determined by high-performance liquid chromatographic (HPLC) system (Dionex, California, USA), equipped with an anionic exchange column (Carbopac PA-100) and a pulsed electrochemical detector. Glucose and xylose were separated with 16 mM sodium hydroxide at a flow rate of 0.25 ml/min, and identified by the respective standards. Fucose was used as an internal standard.

## Additional file

10.1186/s13068-016-0569-z Comparison of seedling growth between control plants (PH and PRV, wild-type and transformed with the empty vector, respectively) and transplastomic plants expressing endoglucanase (DC1, DC2 and DC3) 1 week (A), and 3 weeks after sowing (B) in growth chamber. **Figure S2.** Northern analysis of total RNA isolated from transplastomic and control plants using the *endo* (A), *xyn* (B) and celB (C) coding regions as probes. The scheme (D) show the origin of monocistronic (*) and dicistronic (**) read-through transcripts expected for the different cassettes integrated into the plastid genome, respectively. *goi* = gene of interest. **Figure S3.** Comparison of *rbcL* mRNA accumulation in control (PH) and ß-glucosidase transplastomic (DC21 and DC23) plants. **Figure S4.** Detection of cellulolytic enzymes accumulated in subsequent generations of transplastomic DC plants by Western blot analysis (A-B-C) and Coomassie blue staining of polyacrylamide gel (D–E). Each transplastomic line shows a prominent additional protein band corresponding in size to the foreign protein. DC1, DC2, DC3 = T2 transplastomic plants expressing endoglucanase; DC11 = T2 transplastomic plants expressing endo-β-1,4-xylanase (xyn); DC21, DC23 = T1 transplastomic plants expressing β-glucosidase (celB); PRV = transplastomic control plant transformed with the empty vector; PH = wild-type plant. **Table S1.** Specific enzyme activities measured in crude or enriched extracts of T2 (endo-β-1,4-xylanase) and T1 (β-glucosidase) generations of transplastomic plants.

## Abbreviations

TSP: total soluble proteins
*endo*: *endoglucanase* from *Sulfolobus solfataricus*
*xyn*: *endo-β-1,4*-*xylanase* from *Alicyclobacillus acidocaldarius*
*celB*: *β-glucosidase* from *Pyrococcus furiosus*
5′-UTR: 5′ untranslated region
5′-TCR: 5′ translation control region
*aadA*: *aminoglycoside 3′**adenylyltransferase* gene
MGX: 4-*O*-methyl-glucurono-xylan
pNPG: 4-nitro-phenyl-β-d-glucopyranoside
3′-UTR: 3′ untranslated region
