# Supplementary material for: High-level expression of thermostable cellulolytic enzymes in tobacco transplastomic plants and their use in hydrolysis of an industrially pretreated Arundo donax L. biomass
Source: Biotechnol Biofuels. 2016 Jul 22;9:154. doi: 10.1186/s13068-016-0569-z (PMC4957871; doi:10.1186/s13068-016-0569-z)
Supplement: Supplementary file 1 — 10.1186/s13068-016-0569-z Comparison of seedling growth between control plants (PH and PRV, wild-type and transformed with the empty vector, respectively) and transplastomic plants expressing endoglucanase (DC1, DC2 and DC3) 1 week (A), and 3 weeks after sowing (B) in growth chamber. Figure S2. Northern analysis of total RNA isolated from transplastomic and control plants using the endo (A), xyn (B) and celB (C) coding regions as probes. The scheme (D) show the origin of monocistronic (*) and dicistronic (**) read-through transcripts expected for the different cassettes integrated into the plastid genome, respectively. goi = gene of interest. Figure S3. Comparison of rbcL mRNA accumulation in control (PH) and ß-glucosidase transplastomic (DC21 and DC23) plants. Figure S4. Detection of cellulolytic enzymes accumulated in subsequent generations of transplastomic DC plants by Western blot analysis (A-B-C) and Coomassie blue staining of polyacrylamide gel (D–E). Each transplastomic line shows a prominent additional protein band corresponding in size to the foreign protein. DC1, DC2, DC3 = T2 transplastomic plants expressing endoglucanase; DC11 = T2 transplastomic plants expressing endo-β-1,4-xylanase (xyn); DC21, DC23 = T1 transplastomic plants expressing β-glucosidase (celB); PRV = transplastomic control plant transformed with the empty vector; PH = wild-type plant. Table S1. Specific enzyme activities measured in crude or enriched extracts of T2 (endo-β-1,4-xylanase) and T1 (β-glucosidase) generations of transplastomic plants. [file 13068_2016_569_MOESM1_ESM.pdf]

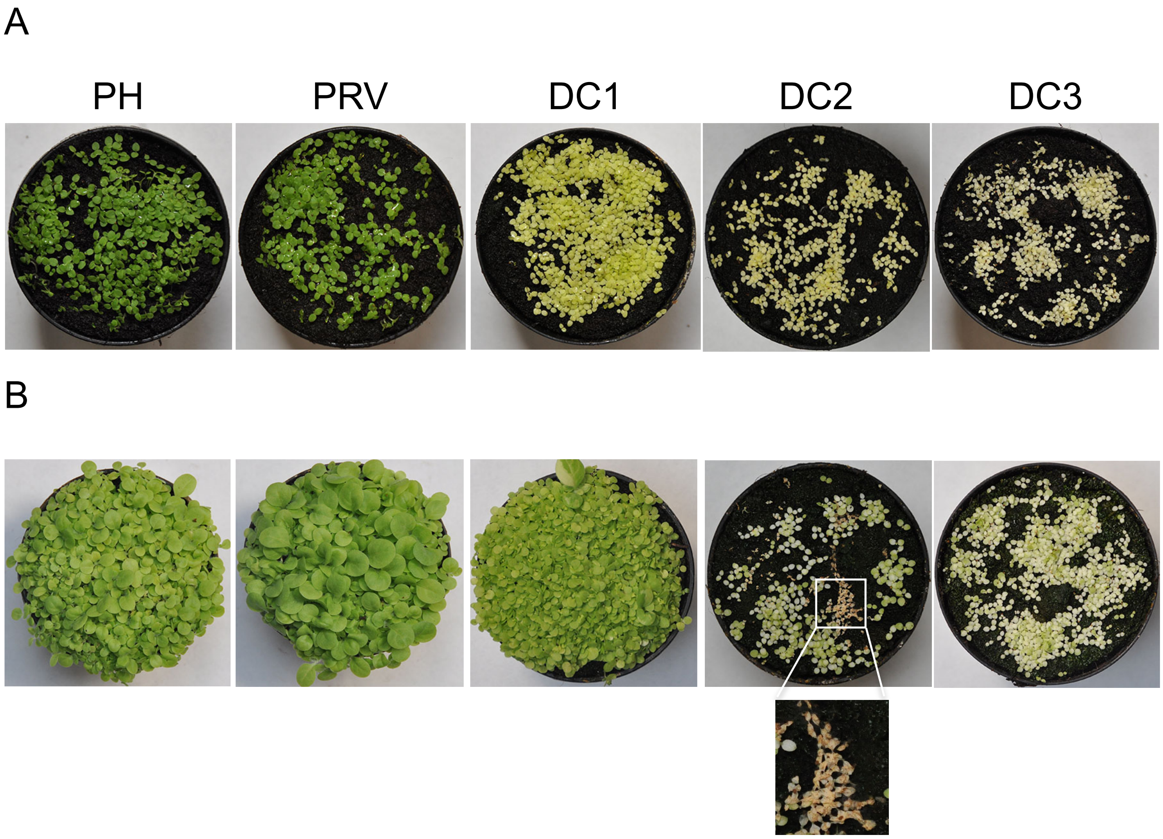

Additional Figure S1 Comparison of seedling growth between control plants (PH and PRV, wild-type and transformed with the empty vector, respectively) and transplastomic plants expressing endoglucanase (DC1, DC2 and DC3) one week (A), and three weeks after sowing (B) in growth chamber.

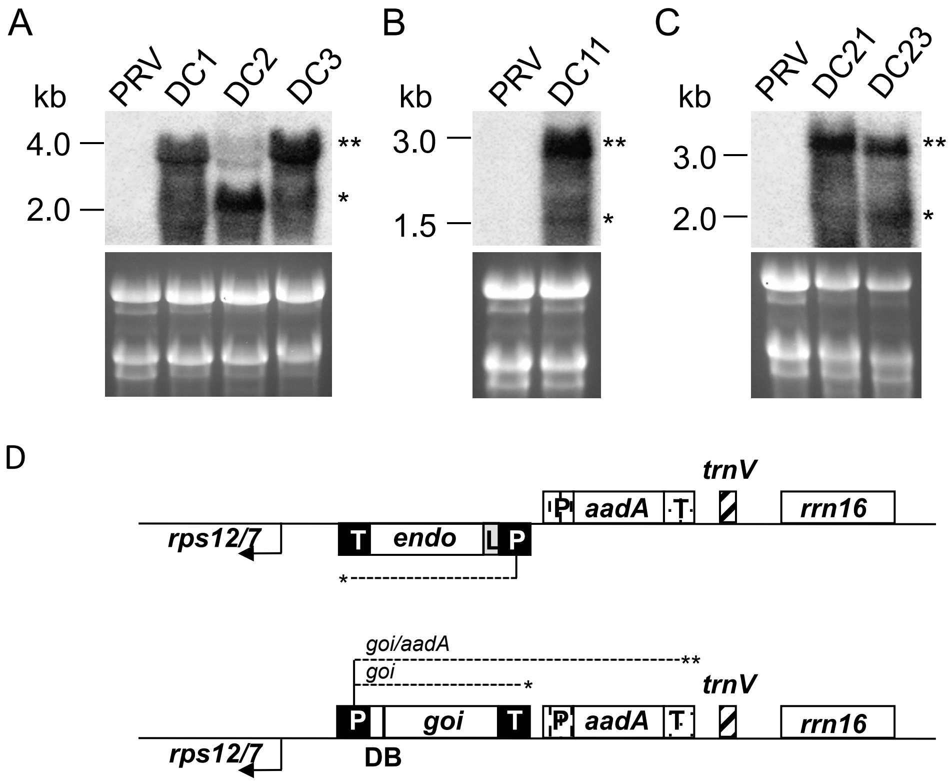

Additional Figure S2 Northern analysis of total RNA isolated from transplastomic and control plants using the *endo* (A), *xyn* (B) and celB (C) coding regions as probes. The scheme (D) show the origin of monocistronic (*) and dicistronic (**) read-through transcripts expected for the different cassettes integrated into the plastid genome, respectively. *goi*= gene of interest.

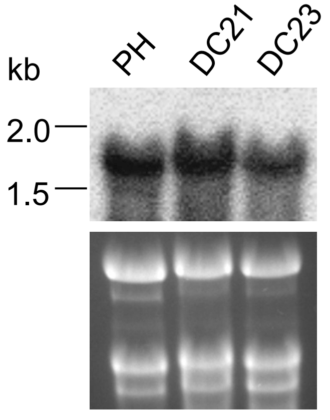

Additional Figure S3 Comparison of *rbcL* mRNA accumulation in control (PH) and β-glucosidase transplastomic (DC21 and DC23) plants.

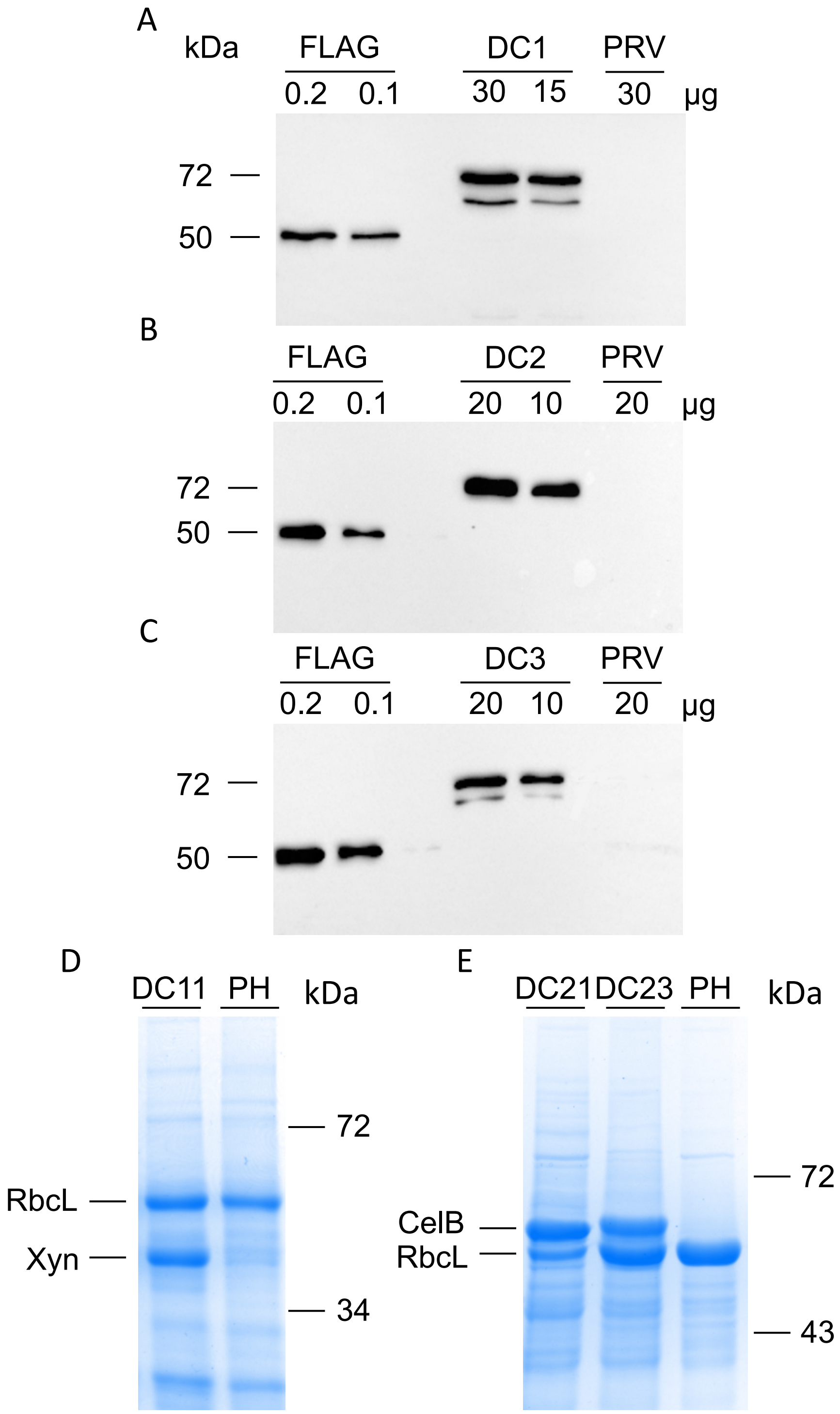

Additional Figure S4 Detection of cellulolytic enzymes accumulated in subsequent generations of transplastomic DC plants by Western blot analysis (A-B-C) and Coomassie blue staining of polyacrylamide gel (D-E). Each transplastomic line shows a prominent additional protein band corresponding in size to the foreign protein. DC1, DC2, DC3= T2 transplastomic plants expressing endoglucanase; DC11= T2 transplastomic plants expressing endo-β-1,4-xylanase (xyn); DC21, DC23= T1 transplastomic plants expressing β-glucosidase (celB); PRV= transplastomic control plant transformed with the empty vector; PH= wild-type plant.

Additional Table S1 Specific enzyme activities measured in crude or enriched extracts of T2 (endo-β-1,4-xylanase) and T1 (β-glucosidase) generations of transplastomic plants

Enzyme	Specific activity[a]		Fold purification
	Crude extracts	Enriched extracts	
	U/mg	U/mg	
endo-β-1,4-xylanase	24.2	101,4	4.2
β-glucosidase	58.4	235,0	4

[a]Determined by enzymatic standard assay.
